# Temporal changes in first-phase ejection fraction during evolution of heart failure with preserved ejection fraction and afterload-induced heart failure in mice

**DOI:** 10.1007/s13105-026-01138-6

**Published:** 2026-02-04

**Authors:** Haotian Gu, Norman Catibog, Yue Zhao, Asjad Visnagri, Philip J. Chowienczyk, Ajay M. Shah, Min Zhang

**Affiliations:** School of Cardiovascular and Metabolic Medicine & Sciences, King’s College London BHF Centre of Research Excellence, 125 Coldharbour Lane, London, SE5 9NU UK

**Keywords:** Echocardiography, Mouse, Ventricular function, Diastole, Ejection fraction

## Abstract

The interplay between systolic and diastolic dysfunction in heart failure with preserved ejection fraction (HFpEF) progression is unclear. First-phase ejection fraction (EF1), a sensitive marker of early systolic function, aids in assessing systolic-diastolic relationships in human hypertension and aortic stenosis. This study examines temporal changes in these relationships in mouse models of HFpEF and elevated afterload. Mouse models of abdominal aortic banding (AAB) and HFpEF (induced by hypertension and high fat feeding) underwent comprehensive serial echocardiography. In AAB, EF1 significantly decreased at week 1 post-surgery (18.8 ± 1.2 vs 24.3 ± 0.8%, p<0.001) compared to controls, with further reduction at week 3 (16.8 ± 0.6%) and week 6 (13.9 ± 0.9%, both p<0.001). EF, global longitudinal strain (GLS) and longitudinal strain rate (LSR) remained unchanged until week 3. Isovolumic relaxation time (IVRT) was the only abnormal index of diastolic function at week 1. In the HFpEF model, EF1 significantly decreased at week 2 (19.1 ± 1.1 vs 25.8 ± 1.0%, p<0.001) compared to controls, while EF, GLS, and LSR were unaltered. At week 3, EF1 decreased further (18.1 ± 0.7%) alongside a significant reduction in GLS (p<0.01), while EF and LSR remained unchanged. IVRT increased early in the HFpEF model, followed by later left atrial (LA) enlargement. EF1, an early marker of systolic impairment, decreases early in HFpEF and afterload-induced dysfunction, accompanied by IVRT prolongation. LA dilatation appears later. These findings highlight the interplay between systolic and diastolic dysfunction in HFpEF progression.

## Introduction

Up to half of all patients with heart failure may have heart failure with preserved ejection fraction (HFpEF), a condition that carries significant morbidity and mortality but its underlying mechanisms remain poorly understood [1]. The occurrence of left ventricular diastolic dysfunction is regarded as a hallmark of HFpEF and is thought to contribute significantly to symptoms [2]. It is generally considered that diastolic dysfunction is the initial cardiac functional abnormality in HFpEF although many studies have noted that patients presenting with HFpEF exhibit some signs of systolic dysfunction, for example a reduction in global longitudinal strain (GLS). Nevertheless, the temporal changes in diastolic and systolic function and the inter-relationship between them during the evolution of the condition are hard to define in humans since most patients already have established HFpEF at presentation or diagnosis. This is an important question both for the understanding of the pathophysiology of the condition and potentially in informing therapeutic approaches.

Our previous work in human with hypertension [3] and aortic stenosis [4] suggested that first-phase ejection fraction (EF1), the proportion of ejection fraction measured up to the time of peak aortic valve flow velocity, provides a sensitive measure of early LV systolic function in cases where overall EF is preserved. A reduction in EF1 with preservation of overall EF is observed when LV contraction is prolonged [3]. Basic research into the biophysics of muscle contraction has identified a potential sensing mechanism whereby a reduction in ventricular contractile force initially leads to a compensatory sustained contraction that may preserve overall EF at the expense of delayed relaxation of the ventricle [5–8]. This provides a mechanism that links changes in systolic and diastolic dysfunction. Based on these considerations, temporal changes in EF1 and other markers of cardiac function should provide insights into the inter-relationship between systolic and diastolic dysfunction during the evolution of HFpEF.

In this study, we investigated this question in a murine model of HFpEF where the time-course of evolution of cardiac dysfunction can be closely monitored by non-invasive methods. We performed high-resolution echocardiography to serially assess cardiac function, with a comprehensive assessment of multiple indices including EF1, other markers of systolic function, and previously validated markers of diastolic dysfunction [9]. We also included a mouse model of increased afterload to distinguish between changes occurring in HFpEF and those due to altered afterload per se.

## Methods

### Animals

Studies were approved by the King’s College London Animal Welfare and Ethical Review Body and performed in accordance with UK Home Office Guidance on the Operation of the Animals (Scientific Procedures) Act, 1986. All animals used were C57BL/6J background. To eliminate the potential impact of sex on the cardiac remodeling in response to pressure overload [10] or deoxycorticosterone acetate (DOCA) treatment [11], only male mice were used in the animal study. All mice were housed under a 12:12-hour light/dark cycle (lights on/off at 7:00/19:00) in specific-pathogen–free conditions. Mice were randomly allocated into following experimental groups. No animals were excluded from this study.

### Experimental model one: Abdominal aortic banding

Abdominal aortic banding (AAB) was used as a well-established model of chronic pressure overload[12]. Briefly, AAB was induced by suprarenal banding under 2% isoflurane anaesthesia, as previously described [13]. The severity of constriction assessed by echo-Doppler 1 week after AAB was similar (constriction degree: 85.2 ± 0.77%, pressure gradient: 17.2 ± 2.8mmHg). Sham constriction as a control group involved identical surgery apart from band placement. Mouse cardiac geometry and function were serially examined by echocardiography at week one, three and six post-surgery.

### Experimental model two: HFpEF

A two-hit mouse model of HFpEF was established involving concomitant (DOCA)/salt-induced hypertension and high fat diet (HFD)-induced metabolic stress. A unilateral nephrectomy was performed in mice aged 10–12 weeks old under i.p. ketamine (75 mg/kg) and medetomidine hydrochloride (1 mg/kg) anaesthesia, followed by subcutaneous implantation of a DOCA pellet (Innovative Research of America, cat. #M-121). Animals also received a 1% NaCl drinking water solution to mimic salt-sensitive hypertension [14, 15]. In addition, mice were fed HFD (D12492, Research Diet) (60% fat, 25.8% protein, 25% carbohydrates). The control group underwent surgery without nephrectomy, DOCA or salt, and were fed with chow diet (2916, Teklad). Mouse cardiac geometry and function were examined by echocardiography at week one, two and three post-surgery.

### Echocardiography

Standard 2D Echocardiography was performed under 1.5% isoflurane anaesthesia at heart rates > 400 bpm, using a Vevo3100 system with a 40 MHz linear probe (Visualsonics, Canada) by an experience operator [13, 16]. The electrocardiography (ECG) was recorded using limb electrodes. Left ventricular diameters, wall thickness, end-diastolic volume (EDV), end-systolic volume (ESV) and EF were measured from the parasternal long-axis view[17].

Diastolic parameters, including the isovolumic relaxation time (IVRT, assessed from mitral Doppler recordings), the E/e′ ratio (mitral inflow E wave/tissue Doppler at the septal corner of the mitral annular velocity e’), and left atrial (LA) area were acquired in the apical 4-chamber view as described previously[9]. To account for the influence of heart rate (HR) on IVRT, a heart-rate–corrected IVRT (IVRTc) was derived according to the formula: IVRTc= $\:\frac{IVRT}{\sqrt{\frac{60}{HR}}}.\:$Speckle tracking was performed in the parasternal long axis view using the Vevo3100 LAB Software 5.7.1 (Visualsonics, Canada) to calculate LV (GLS), longitudinal strain rate (LSR), and reverse longitudinal strain rate (rLSR)[9, 18]. All images were digitally stored in cine loops consisting of 300 frames and analysed off-line in a blinded manner.

### First-phase ejection fraction

**Fig. 1:**
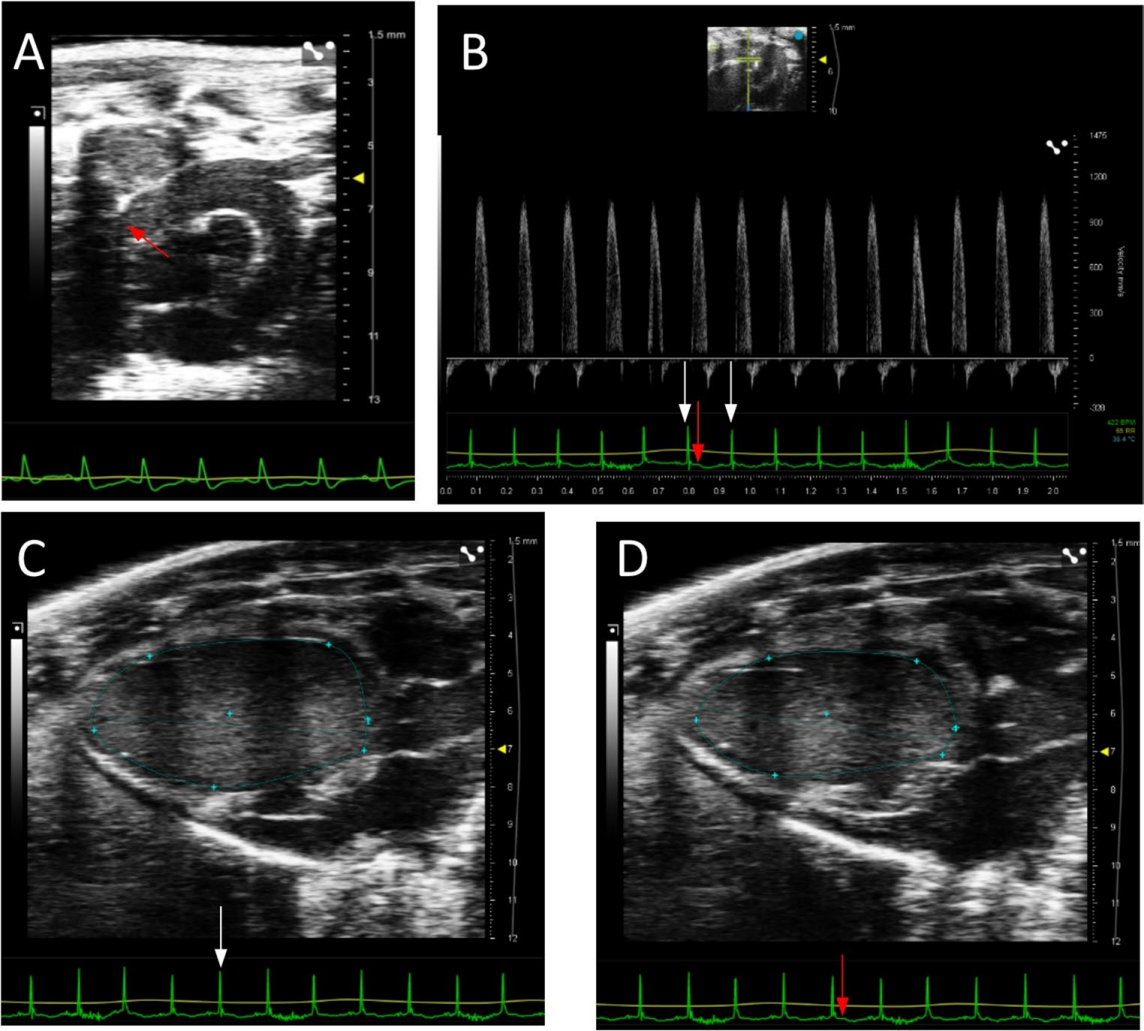
Mouse EF1 measurement by echocardiography. **A**: Aortic flow measurement in high right parasternal view; **B**: Time of peak aortic flow velocity from spectral Doppler (red arrow); **C**: LV end-diastolic volume; **D**: LV volume at the time of peak aortic flow velocity

EF1 was defined as the percentage change in LV volume from end-diastole to the time of peak aortic flow velocity [19]. Aortic flow was acquired in high right parasternal view. Time to peak aortic flow velocity was measured using the Doppler waveform from the ascending aorta just proximal to the aortic valve. EF1 was calculated using the following equation: EF1= (EDV-V1)/EDV%, where V1 is LV volume at time of peak aortic flow velocity (Fig. 1) [3].

### Inter- and intra- observer variability

Intra-observer and inter-observer variability for the parameters of cardiac function were evaluated from 10 randomly selected echocardiographic studies, analysed in a blinded manner on two separate occasions by the same observer (intra-observer variability) or measured independently by two trained operators (inter-observer variability).

### Heart weight and fibrosis measurement

After sacrifice of animals at the end of the study period, the heart was excised, weighed and normalized by the tibia length. Heart tissues were fixed in 4% paraformaldehyde and underwent normal histological preparation into paraffin blocks and onto slides. LV sections were stained with Picrosirius red to assess myocardial interstitial fibrosis. Positive fractional areas were evaluated across the entire section and quantified using ImageJ software [13]. 

### Statistics

Data are presented as mean ± standard error of the mean (SEM). Comparisons between groups were undertaken by unpaired Student’s *t*-test or two-way ANOVA, as appropriate, followed by Tukey post hoc analysis using GraphPad Prism 10.1.2. Data were assessed for normality using the Shapiro-Wilk test. The sample size was selected based on power calculation and previous studies in similar animal models[9, 13], which demonstrated robust findings with similar sample sizes. *P* < 0.05 was considered statistically significant.

## Results

### Experimental model one: Abdominal aortic banding

#### Left ventricular systolic function

**Fig. 2:**
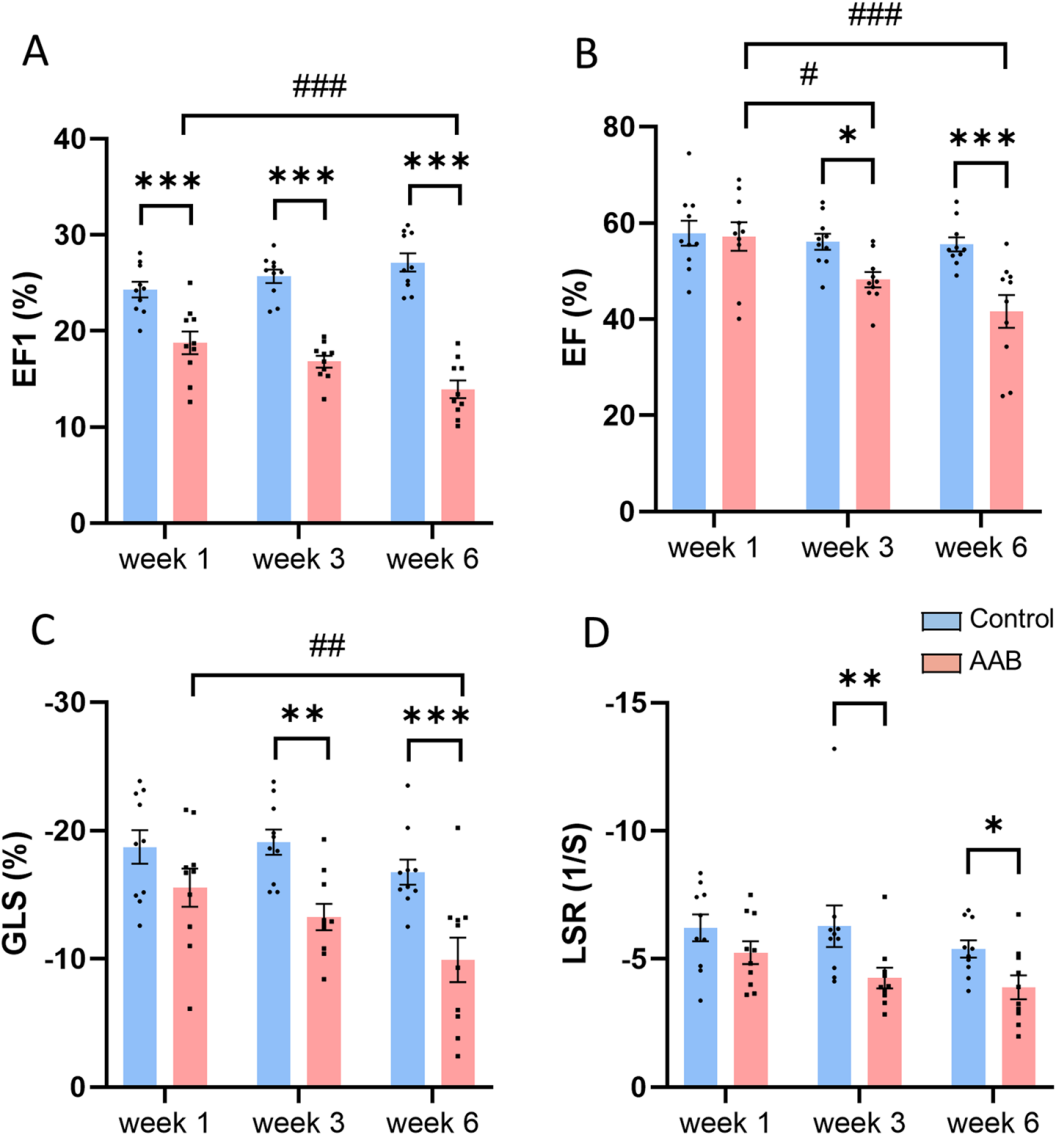
Echocardiographic assessment of LV systolic function in mouse model of pressure overload induced by abdominal aortic banding (AAB). **A**: first-phase ejection fraction (EF1); **B**: ejection fraction (EF); **C**: global longitudinal strain (GLS); **D**: longitudinal strain rate (LSR). *p < 0.05, **p < 0.01, ***p < 0.001, #*p* < 0.05, ##*p* < 0.01, ###*p* < 0.001, *n* = 10/group. Two-way ANOVA with a post-hoc Tukey’s test. All data are presented as the mean ± SEM

In the AAB group, EF1 exhibited a significant decrease at week 1 after the operation (18.8 ± 1.2 vs. 24.3 ± 0.8%, *p* < 0.001) compared to the control group, with a further reduction over the observational period (16.8 ± 0.6 vs. 25.7 ± 0.7% at week 3, 13.9 ± 0.9 vs. 27.1 ± 0.9% at week 6, both *p* < 0.001) (Fig. 2A). Heart rates were similar in the banded and control groups (Table 1). In contrast, at week 1, there was no significant difference in conventional global measures of systolic function, such as EF, GLS, and LSR between the AAB and control group. EF, GLS and LSR started to show a significant reduction at week 3 post-surgery compared to the control group, with further decreases at week 6 (Fig. 2B-D). As expected, AAB was associated with significant increases in LV posterior wall thickness and in LV internal dimensions at end-diastole (Table 1).

**Table 1:** Echocardiographic assessment of cardiac parameters in mouse model of pressure overload induced by abdominal aortic banding (AAB)

	Control	AAB	Control	AAB	control	AAB
	(Week 1)		(Week 3)		(Week 6)	
HR (beats/min)	476 ± 9	445 ± 7	474 ± 18	465 ± 11	453 ± 13	465 ± 12
IVRTc (ms)	41.6 ± 0.9	48.3 ± 1.5 **	42.2 ± 1.9	49.2 ± 1.9 **	43.2 ± 1.2	50.0 ± 1.7 **
LVEDV (µl)	35.5 ± 1.8	36.7 ± 2.4	40.5 ± 2.4	46.7 ± 2.4	39.4 ± 2.4	62.8 ± 8.1 ***, ###, ꝉ
LVESV (µl)	15.0 ± 1.2	15.8 ± 1.5	17.8 ± 1.3	24.3 ± 1.6	17.5 ± 1.2	38.8 ± 7.6 ***, ###, ꝉꝉ
V1 (µl)	26.9 ± 1.4	29.9 ± 2.1	30.1 ± 1.9	38.8 ± 1.9	28.8 ± 2.0	54.3 ± 7.3 ***, ###, ꝉꝉ
IVSd (mm)	0.820 ± 0.024	0.911 ± 0.053	0.835 ± 0.008	1.048 ± 0.039 ***, #	0.848 ± 0.023	1.057 ± 0.048 ***, #
LVIDd (mm)	3.686 ± 0.084	3.713 ± 0.077	3.748 ± 0.039	3.950 ± 0.059	3.774 ± 0.053	4.480 ± 0.157 ***, ###, ꝉꝉ
LVPWd (mm)	0.729 ± 0.022	0.764 ± 0.022	0.741 ± 0.022	0.871 ± 0.041 ***, #	0.812 ± 0.017	0.901 ± 0.024 *, ##

HR, heart rates; IVRTc, isovolumic relaxation time corrected toheart rate; LVEDV, left ventricular end-diastolic volume; LVESV, left ventricular end-systolicvolume; V1, left ventricular volume from end-diastole to the time of peak aortic flow velocity;LVIDd, left ventricular internal dimension at end-diastole; IVSd, intraventricular septumthickness in diastole; LVPWd, end-diastolic left ventricular posterior wall thickness. *p<0.05,***p<0.001 vs respective control groups, #p<0.05, ###p<0.001 vs AAB at week 1, †p<0.05,††p<0.01 vs AAB at week 3, n=10/group. Two-way ANOVA with a post-hoc Tukey’s test. All dataare presented as the mean ± SEM

#### Left ventricular diastolic function

**Fig. 3:**
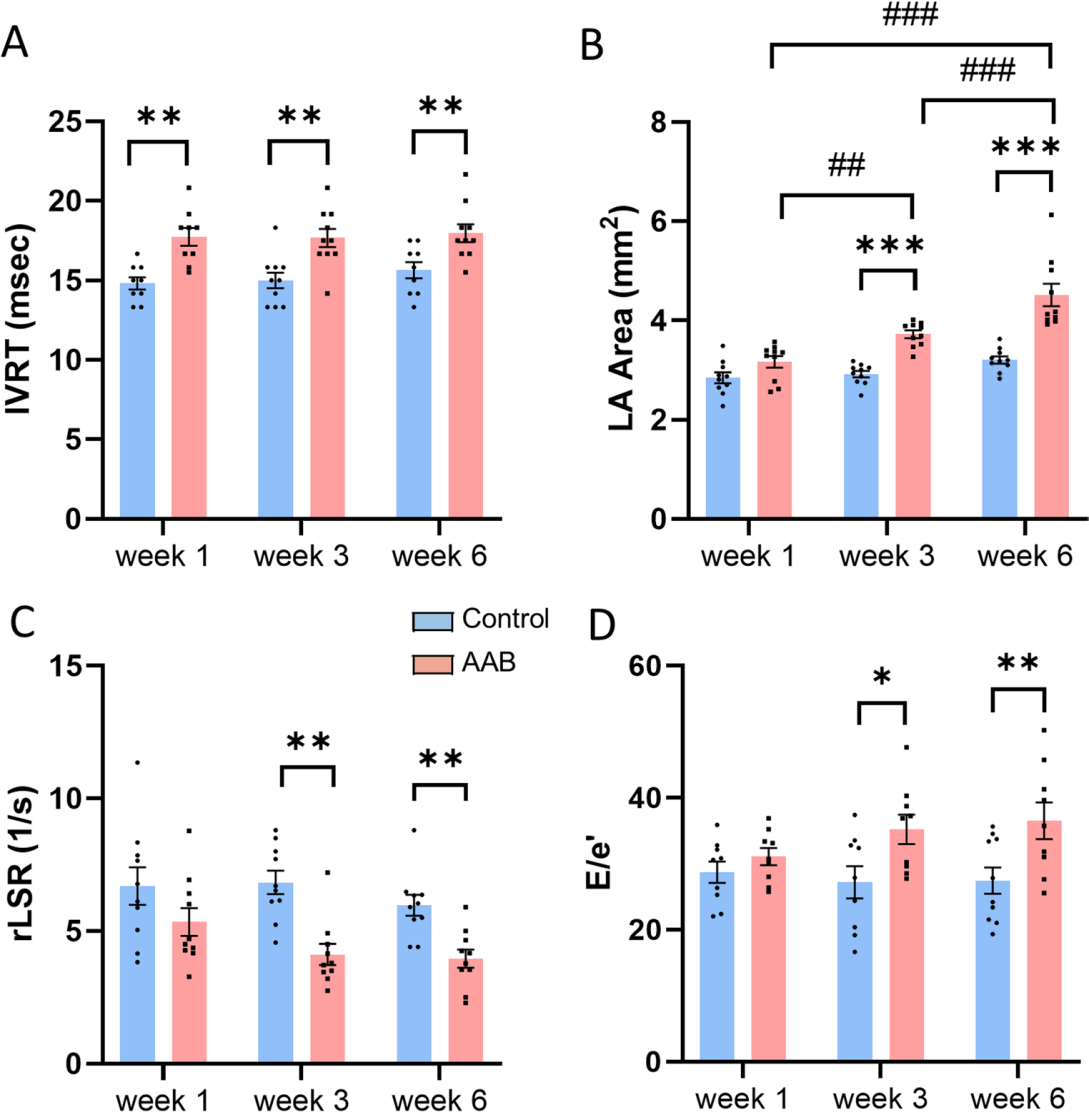
LV diastolic function in mouse model of pressure overload stressed by abdominal aortic banding (AAB). **A**: isovolumetric relaxation time (IVRT); **B**: left atrial (LA) area; **C**: reverse longitudinal strain rate (rLSR); **D**: ratio of early diastolic mitral inflow velocity to early diastolic mitral annulus velocity (E/e’). *p < 0.05, **p < 0.01, ***p < 0.001, ##*p* < 0.01, ###*p* < 0.001, *n* = 10/group. Two-way ANOVA with a post-hoc Tukey’s test. All data are presented as the mean ± SEM

In the AAB group, early signs of impaired relaxation were observed, with a prolonged IVRT compared to the control group at week 1 (17.74 ± 0.57 vs 14.81 ± 0.39 ms, *p* < 0.01). IVRT remained elevated after correction for heart rate (Table 1). However, LA area, rLSR and E/e’ showed no significant differences between the two groups at week 1 (Fig. 3). By week 3, there was a sustained increase in IVRT as well as significant increases in LA area (3.72 ± 0.08 vs 2.92 ± 0.07 mm^2^, *p* < 0.001) and E/e’ (35.2 ± 2.2 vs 27.2 ± 2.5, *p* < 0.05), coupled with a significant decrease in rLSR (4.12 ± 0.40 vs 6.84 ± 0.44 1/s, *p* < 0.01) in the AAB compared to control group (Fig. 3B-D). At week 6, there was clear evidence of LV diastolic dysfunction, reflected by greater increases in E/e’ and LA area, and a further decrease in rLSR (Fig. 3).

### Experimental model two: HFpEF

#### Left ventricular systolic function

**Fig. 4:**
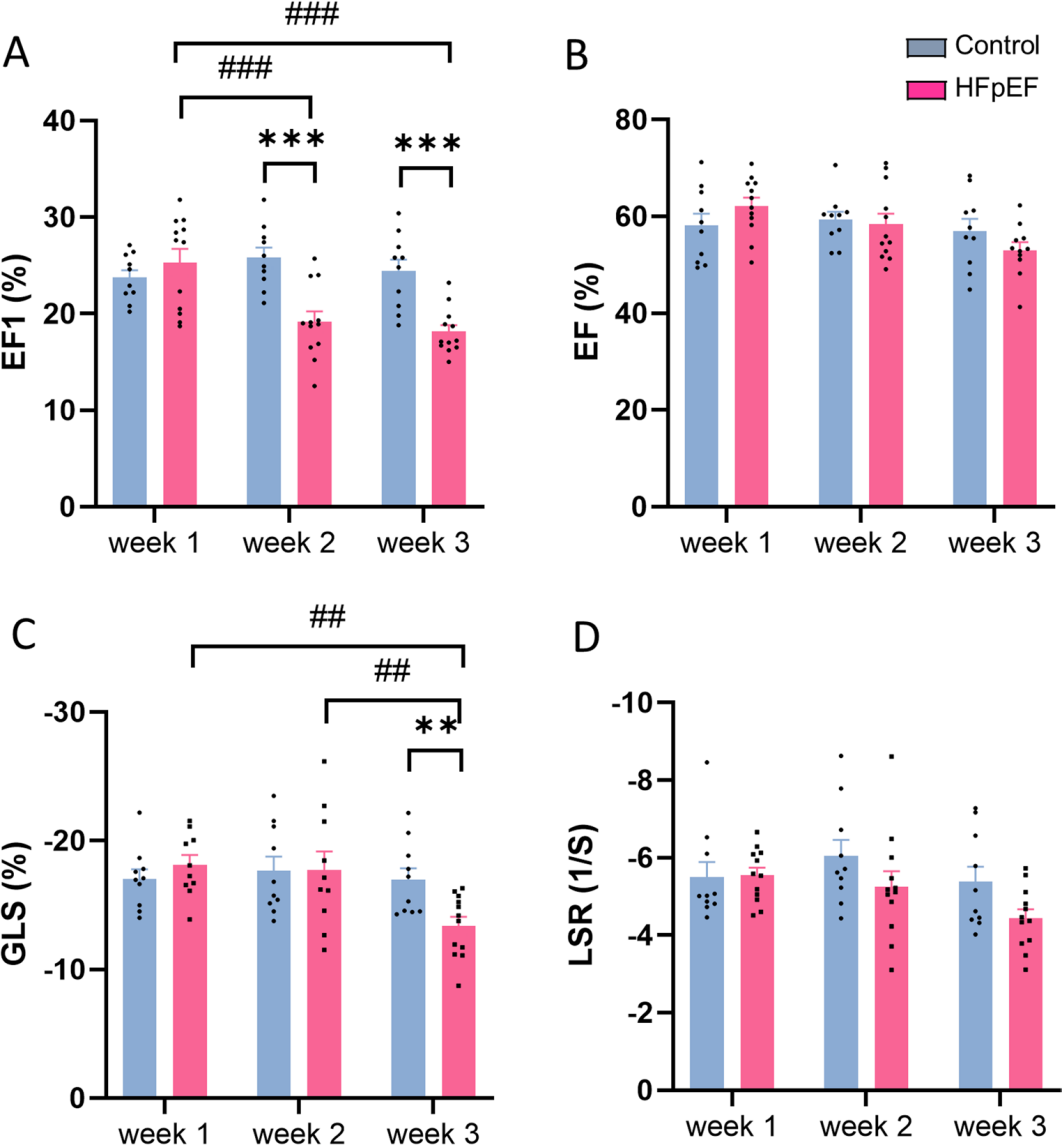
Echocardiographic assessment of LV systolic function in mouse model of heart failure with preserved ejection fraction (HFpEF). **A**: first-phase ejection fraction (EF1); **B**: ejection fraction (EF); **C**: global longitudinal strain (GLS); **D**: longitudinal strain rate (LSR). **p < 0.01, ***p < 0.001, ##*p* < 0.01, ###*p* < 0.001, *n* = 10–12/group. Two-way ANOVA with a post-hoc Tukey’s test. All data are presented as the mean ± SEM

In the HFpEF group, EF1 exhibited a significant decrease compared to the control group at week 2 (19.1 ± 1.1 vs. 25.8 ± 1.0%, *p* < 0.001) (Fig. 4A). There was no reduction in EF or LSR at any point in this model while a decrease in GLS was observed at 3 weeks (Fig. 4B-D).

#### Left ventricular diastolic function

**Fig. 5:**
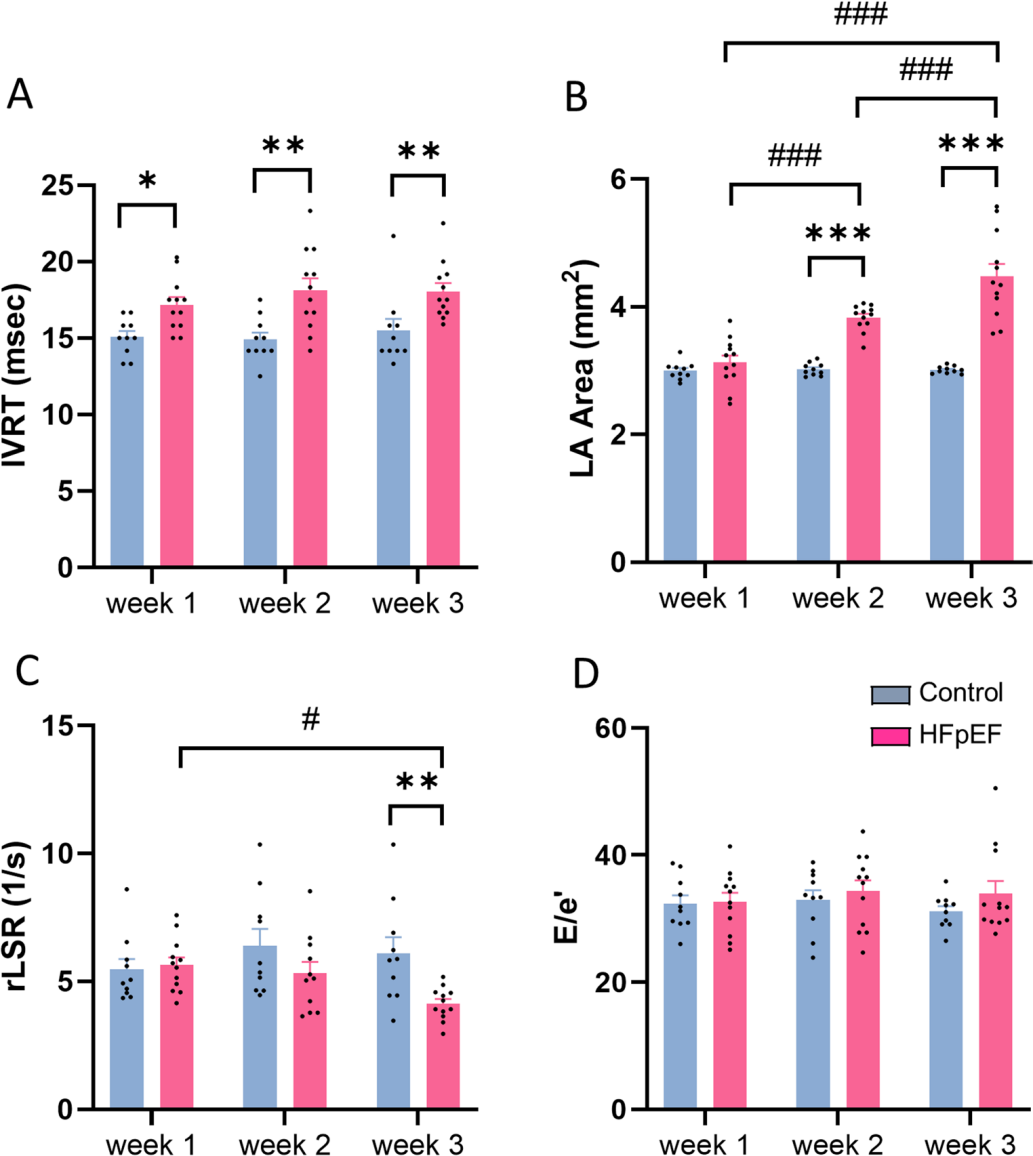
LV diastolic function in mouse model of heart failure with preserved ejection fraction (HFpEF). **A**: isovolumetric relaxation time (IVRT); **B**: left atrial (LA) area; **C**: reverse longitudinal strain rate (rLSR); **D**: ratio of early diastolic mitral inflow velocity to early diastolic mitral annulus velocity (E/e’). *p < 0.05, **p < 0.01, #*p* < 0.05, ###*p* < 0.001, *n* = 10–12/group. Two-way ANOVA with a post-hoc Tukey’s test. All data are presented as the mean ± SEM

In the HFpEF group, early signs of impaired relaxation were observed, indicated by prolonged IVRT compared to the control group (17.18 ± 0.50 vs 15.08 ± 0.38ms, *p* < 0.05) at week 1 (Fig. 5A). This difference persisted after correction for heart rate (Table 2). LA area, rLSR and E/e’ showed no significant differences between the two groups at week 1 (Fig. 5B-D).

**Table 2:** Echocardiographic assessment of cardiac parameters in mouse model of heart failure with preserved ejection fraction (HFpEF)

	Control	HFpEF	Control	HFpEF	Control	HFpEF
	(Week 1)		(Week2)		(Week 3)	
HR (beats/min)	436 ± 10	446 ± 11	453 ± 16	427 ± 9	446 ± 8	428 ± 5
IVRTc (ms)	40.7 ± 1.4	46.7 ± 1.1 **	40.8 ± 0.9	48.2 ± 1.9 **	42.2 ± 2.0	48.3 ± 1.7 **
LVEDV (µl)	46.4 ± 1.8	46.5 ± 2.7	45.3 ± 2.8	50.3 ± 2.0	50.0 ± 2.5	53.7 ± 2.3
LVESV (µl)	19.6 ± 1.6	17.8 ± 1.7	18.4 ± 1.2	21.2 ± 1.6	21.7 ± 1.8	26.0 ± 1.7 ##
V1 (µl)	35.4 ± 1.3	34.8 ± 2.2	33.6 ± 2.2	40.8 ± 1.8 *	37.8 ± 2.0	44.0 ± 2.0 *, ##
IVSd (mm)	0.768 ± 0.017	0.844 ± 0.033	0.802 ± 0.030	0.900 ± 0.026 *	0.848 ± 0.020	0.990 ± 0.032 ***, ###
LVIDd (mm)	3.902 ± 0.124	3.880 ± 0.087	3.903 ± 0.110	3.999 ± 0.057	4.058 ± 0.095	4.250 ± 0.105 #
LVPWd (mm)	0.806 ± 0.057	0.792 ± 0.021	0.834 ± 0.042	0.816 ± 0.020	0.848 ± 0.023	0.903 ± 0.021 #

HR, heart rates; IVRTc, isovolumic relaxation time corrected to heartrate; LVEDV, left ventricular end-diastolic volume; LVESV, left ventricular end-systolic volume; V1,left ventricular volume from end-diastole to the time of peak aortic flow velocity; LVIDd, leftventricular internal dimension at end-diastole; IVSd, intraventricular septum thickness in diastole; LVPWd, end-diastolic left ventricular posterior wall thickness. *p<0.05, ***p<0.001 vs respective control groups, #p<0.05, ##p<0.01, ###p<0.001vs HFpEF at week 1, n=10-12/group. Two-way ANOVA with a post-hoc Tukey’s test. All data are presented as the mean ± SEM

At week 2, there was evidence of a further increase in IVRT (18.13 ± 0.78ms) accompanied by a significant enlargement of LA area (3.83 ± 0.06 vs 3.02 ± 0.03mm^2^, *p* < 0.001) in the HFpEF compared to control group (Fig. 5A and B). At week 3, LV diastolic dysfunction became more apparent, as evidenced by further increases in IVRT and LA area, coupled with a significant decrease in rLSR (4.13 ± 0.18 vs 6.10 ± 0.64 1/s, *p* < 0.01) (Fig. 5A-C); however, E/e’ was not significantly altered (Fig. 5D).

The inter- and intra-observer variability for diastolic parameters are shown in Table 3.

**Table 3:** Intra- and interobserver coefficient of variation of main functional parameters evaluated by echocardiography

Parameter	Intra-observer coefficient of variation (%)	Inter-observer coefficient of variation (%)
EF	2.29	2.43
EF1	3.79	5.25
GLS	8.05	6.61
LSR	2.76	2.62
IVRT	1.26	3.94
LAA	3.50	3.41
rLSR	1.57	1.69
E/e’	6.4	9.47

*EF* ejection fraction, *EF1* first-phase ejection fraction, *GLS* global longitudinal strain, *LSR* longitudinal strain rate, *IVRT* isovolumetric relaxation time, *LAA* left atrial area, *rLSR* reverse longitudinal strain rate, *E/e’* ratio of early diastolic mitral inflow velocity to early diastolic mitral annulus velocity

#### LV mass and myocardial fibrosis

**Fig. 6:**
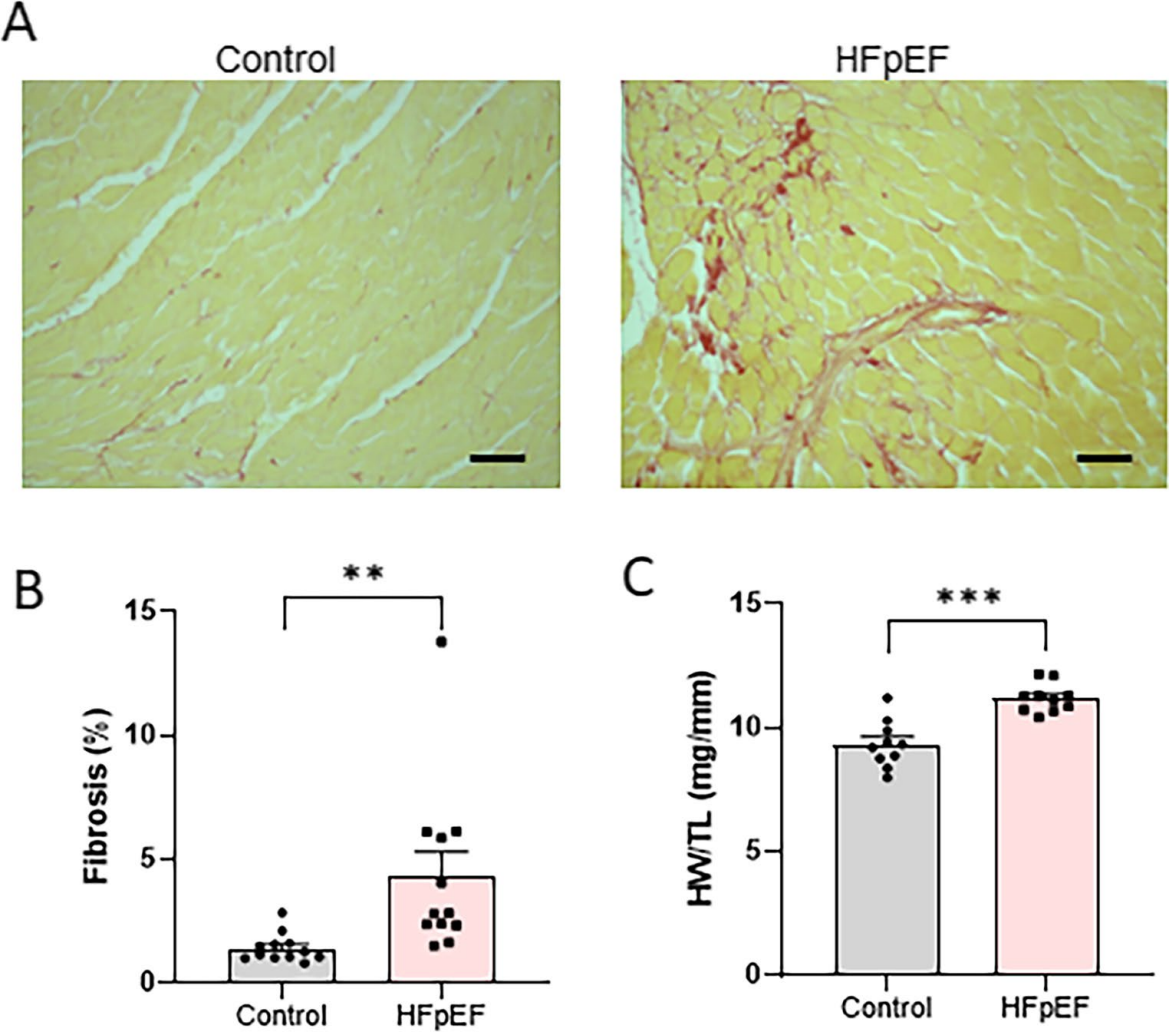
Myocardial fibrosis and cardiac hypertrophy in mouse model of heart failure with preserved ejection fraction (HFpEF). A: representative images with Picrosirius red staining. scale bar: 50 μm; B: quantification of myocardial interstitial fibrosis; C: heart weight (HW) to tibia length (TL) ratio. **p < 0.01, ***p < 0.001, *n* = 10–12/group. Unpaired t-test. All data are presented as the mean ± SEM

At the end of the observational period, mice in the HFpEF group exhibited a significant increase in myocardial interstitial collagen fibres by Picrosirius red staining and cardiac hypertrophy in terms of elevated heart weight/tibia length ratio compared to those in the control group (Fig. 6). Echocardiography also revealed significant increases in intraventricular septum thickness and LV posterior wall thickness at end-diastole in HFpEF group (Table 2).

## Discussion

Our study assessed EF1, a sensitive measure of LV early systolic function, in experimental mouse models. The main findings are: (1) EF1 can be reliably measured in the mouse by echocardiography using a 40 MHz linear probe; (2) EF1 demonstrates greater sensitivity in detecting early LV systolic impairment than conventional systolic function measures such as EF, GLS and LSR, both in pressure overload and HFpEF mouse models; (3) early reduction of EF1 in HFpEF parallels changes in LV relaxation. These findings align with human studies where lower EF1 is linked to deteriorating diastolic function in patients with hypertension [3]. 

EF1, as a sensitive measure of left ventricular contraction during the early systolic phase, offers valuable insights into cardiac function. In certain conditions, an impairment of myocardial contractile function may be masked by a more prolonged (sustained) contraction through systole, preserving overall EF. Traditional markers such as EF and GLS assess overall contraction across the entire systolic period, potentially overlooking early-phase dysfunction. EF1 provides a distinct focus on early systolic function, offering a nuanced perspective beyond global systolic measures. While EF and GLS assess global contraction over the entire systolic period, EF1 captures subtle changes that might be missed by broader metrics. This specificity is crucial in uncovering early dysfunction that might not be evident when relying solely on global systolic measures.

In the AAB model, EF1 displayed a significant early decrease at week 1, persisting and progressing through weeks 3 and 6. This contrasts with conventional measures such as EF, GLS, and LSR, which exhibited significant reductions only starting at week 3. Conversely, in the HFpEF model, EF1 reduction occurred later (week 2) but exhibited a more abrupt response at week 2, suggesting a different temporal pattern compared to the AAB model.

In both models, conventional measures (such as EF, GLS, and LSR) showed significant alterations, but their changes manifest later compared to EF1. The earlier sensitivity of EF1 complements these conventional measures, providing a novel and more comprehensive understanding of the dynamics of systolic function over time. This early response aligns with the notion that EF1 is uniquely positioned to both capture subtle changes in cardiac contraction during the initial phase of systole and the interaction between systolic function and relaxation.

Mice in both groups exhibited early signs of impaired ventricular relaxation characterized by prolonged IVRT while enlargement of LA area, which relates to increases in LV end-diastolic pressure, occurred later. The data are consistent with the notion that early diastolic impairment in both models may be related to functional factors (such as impaired myocardial relaxation due to metabolic defects [20] or disrupted calcium handling [21]), whereas the later stages of impairment may be more related to structural factors such as interstitial fibrosis (which increases ventricular stiffness). We noted that the HFpEF group exhibit a more rapid progression to diastolic dysfunction than the AAB group which may reflect differences in underlying pathophysiology. Interestingly, E/e’, a commonly used marker for the diagnosis of diastolic dysfunction in patients, is not significantly changed in the mouse model of HFpEF. This finding is consistent with our previous study [9]. 

A notable finding in this study is that early systolic impairment detected by EF1 parallels changes in diastolic function, as is also the case in humans [3]. These findings underscore the potential of EF1 as a bridging metric that provides a more integrated view of cardiac systolic and diastolic function. Traditional measures often compartmentalise systolic and diastolic assessments, potentially missing interconnected dynamics. EF1, by revealing subtle systolic alterations and their temporal relationship with changes in relaxation, offers a comprehensive narrative of cardiac function. The observed patterns in mouse models and how they parallel changes documented in humans suggest that EF1 holds promise as a translatable measure for use in studies that investigate pathophysiological and haemodynamic mechanisms in cardiovascular diseases.

### Novelty and clinical implications

Several clinical studies have demonstrated that EF1 is a sensitive marker of early systolic dysfunction in patients with hypertension and aortic stenosis, outperforming conventional indices such as EF and GSL. Moreover, EF1 has been shown to be a robust predictor of adverse clinical events across diverse cardiac conditions, highlighting its potential utility for risk stratification and clinical decision-making [4, 22–27]. Our findings extend these clinical observations to mouse models, showing that EF1 reduction represents an early and progressive feature of both pressure overload and HFpEF. These results provide a translational bridge between preclinical pathophysiology and clinical manifestations, reinforcing the relevance of EF1 as a biomarker of evolving cardiac dysfunction.

### Limitations

Our study has several important limitations: (1) only male mice were used to reduce the variability linked to sex differences. Given the higher prevalence of HFpEF in women, further studies in both males and females are needed to determine the generalizability of the findings; (2) we have not included invasive haemodynamic data, as the primary aim of this study was to serially track disease progression especially the subtle and early changes in the two mouse models using non-invasive techniques. Additionally, PV loop analysis is a terminal procedure which can only be performed at the endpoint of the animal model, which adds the limited value to the findings of this study; (3) the baseline phenotypes of the HFpEF mouse model warrant further characterisation, including evaluations of blood pressure, metabolic alterations, and pulmonary edema; (4) mice have fast heart rates, different myocardial structures, and pathophysiologies. Moreover, the development of HF in the murine models used in this study occurs relatively quickly. Thus, caution is also warranted when extrapolating these findings to clinical applications.

## Conclusions

In summary, EF1 emerges as a sensitive and early indicator of systolic impairment in pressure overload and HFpEF mouse models. While this study primarily provides descriptive insights, the temporal changes observed in EF1 alongside alterations in diastolic indices suggest that EF1 may offer valuable information on evolving systo-diastolic dysfunction. Further mechanistic investigations are warranted to fully elucidate its role in the progression of cardiac dysfunction and structural remodeling.

## Data Availability

Data can be made available upon reasonable request from the corresponding author.
